# A comprehensive review of venous thromboembolism risk assessment models for hospitalized medical patients: comparative evidence, implementation challenges, and future directions

**DOI:** 10.3389/fcvm.2025.1738139

**Published:** 2026-01-12

**Authors:** Lama Alfehaid, Nada Alsuhebany, Yahya M. K. Tawfik, Shuroug A. Alowais, Shazia Adnan, Joud Alfriah, Lolwa Alabdelmuhsin, Abdulmajeed M. Alshehri, Majed Alyami

**Affiliations:** 1 Department of Pharmacy Practice, College of Pharmacy, King Saud bin Abdulaziz University for Health Sciences, Riyadh, Saudi Arabia; 2 Department of Pharmaceutical Care Services, King Abdulaziz Medical City, Ministry of National Guard-Health Affairs, Riyadh, Saudi Arabia; 3 King Abdullah International Medical Research Center, Riyadh, Saudi Arabia; 4 Department of Clinical Pharmacy, College of Pharmacy, King Saud University, Riyadh, Saudi Arabia; 5 College of Pharmacy, Princess Nora Bint Abdul Rahman University, Riyadh, Saudi Arabia

**Keywords:** clinical decision support, medical inpatients, risk assessment, thromboprophylaxis, venous thromboembolism

## Abstract

Venous thromboembolism (VTE) is a leading cause of preventable hospital-acquired morbidity and mortality. Despite the availability of effective prophylaxis, its application in clinical practice remains inconsistent, often due to uncertainty in risk stratification. This review evaluates the validity and implementation of VTE risk assessment models (RAMs) in medical inpatients. Seven widely used RAMs, Caprini, Padua, IMPROVE, IMPROVEDD, Wells, Geneva, and Kucher e-alert, are critically examined alongside emerging digital and biomarker-enhanced tools. The Padua and IMPROVE scores show consistent reliability across various medical populations, while the Caprini RAM remains the most accurate in surgical contexts. The Wells deep vein thrombosis (DVT) and revised Geneva scores are preferred for diagnosing suspected thrombosis and pulmonary embolism, respectively. Electronic alerts, such as the Kucher and Woller models, have shown promise in increasing prophylaxis adherence and reducing symptomatic VTE events. Nonetheless, challenges like limited external validation, gaps in clinician training, and inconsistent local protocols hinder their real-world application. Future research should recalibrate RAMs for underrepresented groups, incorporate biomarkers and mobility data, and create user-friendly AI tools that can optimize the balance between thrombosis and bleeding risks. The adoption of validated, user-friendly RAMs is essential for improving thromboprophylaxis, enhancing patient safety, and reducing the burden of hospital-associated VTE.

## 1 Introduction

Venous Thromboembolism (VTE), which includes pulmonary embolism (PE), deep vein thrombosis (DVT), or both, represents a significant healthcare concern. Among hospitalized patients, VTE is recognized as a leading cause of preventable morbidity and mortality, primarily due to complications such as recurrent thrombosis, post-thrombotic syndrome, and chronic thromboembolic pulmonary hypertension (1, 2). Globally, the incidence of VTE is estimated to range from 0.2 to 2 cases per 1,000 person-years, highlighting its widespread impact (3). The societal and economic burden posed by VTE is considerable. It contributes substantially to healthcare costs, including hospitalization, ongoing medical management, and associated disabilities. Recent estimates suggest a disability-adjusted life-year (DALY) rate of approximately 7.6 per 100,000 population, underscoring its significant impact on public health (4). Multiple factors elevate the risk of developing VTE, including acute medical illness, obesity (high body mass index), prolonged immobility, active malignancy, inflammatory conditions, and the use of estrogen-containing medications such as oral contraceptives and hormone replacement therapies (5, 6). Enhanced recognition and understanding of these risk factors are crucial for effective prevention, timely diagnosis, and improved clinical outcomes for patients at risk for VTE.

International guidelines, including the American Society of Hematology (ASH) clinical practice guideline for hospitalized medical patients (7), the UK National Institute for Health and Care Excellence (NICE) guideline NG89 (8), and the American College of Physicians (ACP) guideline, all emphasize that structured risk assessment is the crucial first step in preventing hospital-acquired DVT and PE (9). In response, several risk-assessment models (RAMs), including the Caprini, Padua, Wells, and Kucher scores, have been developed to classify inpatients into low-to-high VTE risk and to guide thromboprophylaxis (10–13). Although validated across diverse surgical and medical cohorts, these instruments exhibit variable performance due to heterogeneity in study populations, inclusion criteria, and outcome definitions, which can compromise sensitivity and limit generalizability (14).

Accordingly, this review examines VTE RAMs across clinical settings, with a primary focus on their applicability to hospitalized medical patients. Evidence from surgical, obstetric, and oncology populations is included where relevant to provide contextual comparison and to clarify the strengths, limitations, and generalizability of individual RAMs when applied to medical inpatients. Special attention is given to populations in whom VTE risk assessment is particularly complex, including patients with active malignancy, pregnancy and the puerperium, and other under-represented inpatient groups. In addition to established clinical RAMs, this review discusses emerging approaches to enhance risk stratification, including biomarker-augmented models and artificial intelligence-based prediction tools. Finally, we address practical implementation challenges, including integration into electronic health records, clinician adoption, and the balance between thrombosis prevention and bleeding risk, and outline future directions for improving personalized VTE prevention in hospitalized patients.

## 2 Methods

This narrative review synthesized and critically appraised evidence on VTE RAMs relevant to hospitalized medical patients, including Caprini, Padua, IMPROVE/IMPROVEDD, Wells, Geneva, and electronic alert tools, with attention to implementation and special populations.

### 2.1 Information sources & search strategy

This review examines VTE RAMs across clinical settings, with a primary focus on their applicability to hospitalized medical patients. Evidence from surgical, obstetric, oncology, pediatrics, and geriatrics populations is included where relevant to provide contextual comparison and to highlight the strengths, limitations, and generalizability of individual models when applied to medical inpatients. PubMed, Scopus, Web of Science, and Google Scholar were searched from inception through September 2025 using MeSH and free-text terms for VTE, deep vein thrombosis, pulmonary embolism, risk assessment, hospitalized medical patients, thromboprophylaxis, and clinical decision support. Reference lists of key articles and guidelines were hand-searched to identify additional studies.

### 2.2 Eligibility criteria

Included sources comprised primary studies, validation/derivation studies, comparative evaluations, implementation studies, and clinical guidelines addressing VTE RAMs in adult medical inpatients. Articles limited to purely surgical, pediatric, or outpatient settings were excluded unless they provided essential comparative context or model adaptations for inpatients.

### 2.3 Selection & data handling

Screening and full-text review were performed by the lead author, with targeted secondary verification for key data elements as needed. Extracted items included study design, setting/population, RAM components, validation metrics (e.g., AUC, sensitivity, specificity), clinical context, and implementation outcomes (e.g., uptake, effect on prophylaxis/VTE).

### 2.4 Synthesis approach

Evidence was organized thematically (historical development, validation performance, comparative accuracy, clinical utility/limitations, and implementation). Dedicated subsections summarized applications in oncology, pregnancy, and emerging biomarker/AI-enabled approaches. No meta-analysis or quantitative pooling was undertaken, consistent with a narrative review objective.

### 2.5 Ethics

This review synthesized publicly available literature and did not involve human subjects or patient-level data; therefore, institutional review board approval was not required.

We synthesized the evidence narratively. Below, we summarize established RAMs, their validation and implementation data, followed by special populations and emerging biomarker/AI approaches.

## 3 Established clinical risk-assessment models

**Figure 1:**
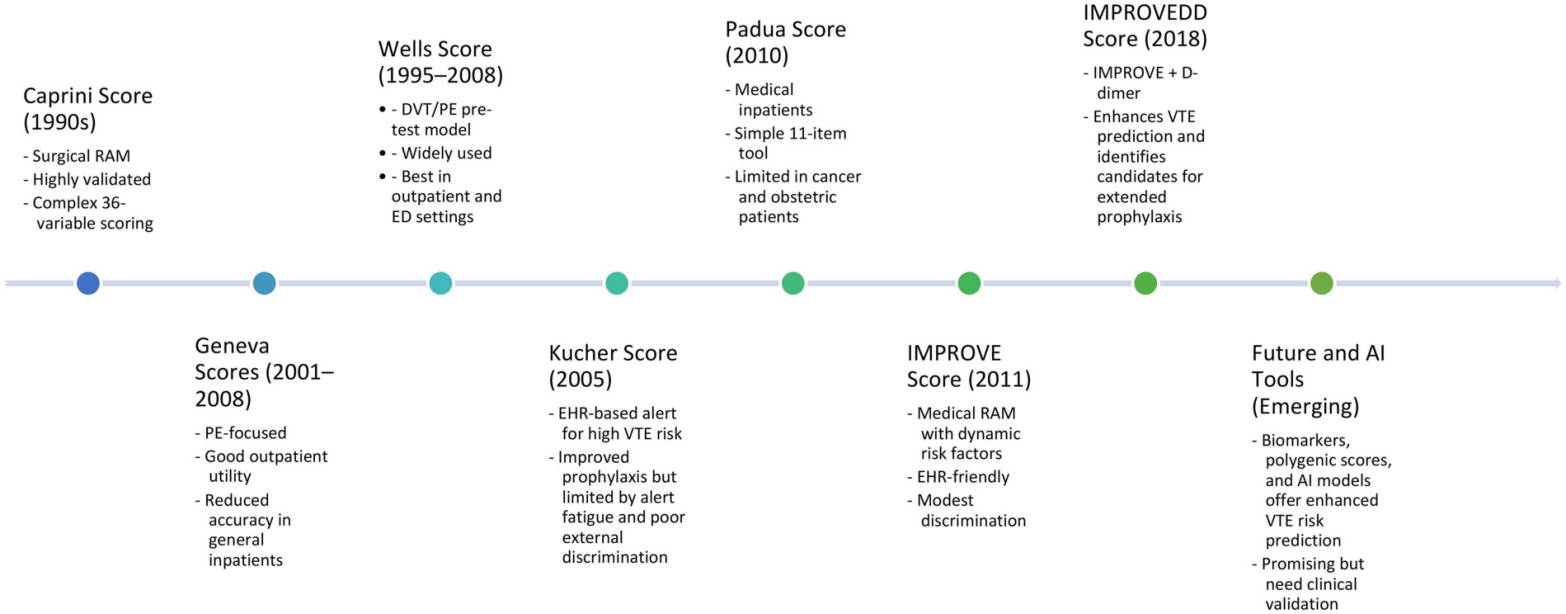
Evolution of clinical and emerging approaches to VTE RAM. Timeline illustrating the development of established clinical RAMs, biomarker-augmented tools, and emerging AI–based approaches for VTE risk stratification. The figure summarizes key conceptual milestones rather than primary datasets and is adapted from original descriptions of the Caprini model (10, 15), PPS (11, 16, 17), IMPROVE (18–20), and IMPROVEDD (21, 22) models and diagnostic Wells (12, 23–27) and Geneva scores (28–31), as well as contemporary reviews of biomarker- and AI-driven approaches (32–37).

A variety of clinical RAMs have been developed over the past three decades to quantify VTE risk across diverse inpatient populations. These tools vary in complexity, target populations, and validation strength, yet collectively form the backbone of contemporary VTE prophylaxis strategies. Figure 1 provides a chronological overview of key RAMs, highlighting their development timelines, key features, and clinical applications. The following sections provide a detailed summary of each model's design, clinical performance, and limitations.

### 3.1 Caprini RAM

#### 3.1.1 Development, validation, and scoring system

The Caprini RAM was originally developed and extensively validated for surgical patients, and its strongest predictive performance has consistently been demonstrated in perioperative and postoperative settings. The Caprini RAM, introduced by Joseph Caprini in the early 1990s, remains the most extensively validated tool for estimating the risk of perioperative VTE. It assigns weighted points to evidence-based risk factors, including patient age, prior VTE, cancer status, and the type and duration of surgery, to generate an aggregate score that guides prophylaxis decisions. The original 20-item version, validated in general surgical cohorts, stratifies patients into low (0–1), moderate (2–4), and high risk (≥5) categories for postoperative VTE (10). Subsequent updates expanded the inventory to 36 variables applicable across multiple surgical specialties. Added factors include varicose veins, inflammatory bowel disease, morbid obesity, cardiorespiratory disease, insulin-dependent diabetes, exposure to hormonal or chemotherapy agents, recurrent pregnancy loss, recent blood transfusion, and smoking history (15).

#### 3.1.2 Clinical implications and limitations

Across surgical populations, the Caprini score exhibits robust diagnostic accuracy, with a sensitivity of approximately 96%, a specificity of approximately 92%, and positive and negative predictive values of roughly 93% and 95.5%, respectively (38), establishing it as a key element of personalized prophylaxis strategies. Its widespread use, however, is limited by the necessity to assess 36 individual variables (15), the lack of universally recognized risk-category thresholds, and a scarcity of validation data in medical or pediatric inpatient settings.

### 3.2 Padua prediction score

#### 3.2.1 Development, validation, and scoring system

The Padua Prediction Score (PPS) was specifically developed to assess VTE risk in acutely ill hospitalized medical patients and is not intended for routine use in surgical or obstetric populations. It was introduced by Barbar and colleagues in 2010 to stratify acutely ill medical inpatients based on 11 weighted clinical variables (total score: 0–20); a score of 4 or higher denotes a high thrombotic risk. In the 1,180-patient derivation cohort, omission of prophylaxis in the high-risk stratum translated into an approximately 11-fold increase in symptomatic VTE, underscoring the model's discriminatory capacity (11). External validations, however, have yielded heterogeneous results. In septic medical patients, the PPS failed to predict in-hospital or one-year VTE, although higher scores were independently associated with inpatient mortality (39). Comparative surgical data suggest the score retains good discrimination for postoperative events (38). Among lung-cancer in-patients receiving immune checkpoint inhibitors, the PPS performs on par with the Caprini model (40). Conversely, studies in obstetric and peripartum cohorts reveal poor sensitivity, highlighting the paucity of validation in this population (41).

The Padua RAM scoring system comprises 11 risk factors for VTE, with a total score ranging from 0 to 20 points, depending on the number and weight of the identified risk factors (Table 1). Patients with a score of 4 or higher are classified as high-risk, while those with a score below four are classified as low-risk (11).

**Table 1:** Padua prediction score.

Weighted score	Risk factors
3 points	• Active cancer • Previous VTE (excluding superficial thrombosis) • Reduced mobility for ≥3 days
2 points	• Recent trauma or surgery (within 1 month)
1 point	• Age ≥70 years • Heart or respiratory failure • Acute myocardial infarction or ischemic stroke • Acute infection or rheumatologic disorder • Obesity (BMI ≥30 kg/m²) • Ongoing hormonal therapy

Components and scoring of the Padua Prediction Score used for VTE risk assessment in acutely ill hospitalized medical patients. Adapted from the original Padua Prediction Score derivation and validation studies and guideline summaries (11).

#### 3.2.2 Clinical implications and limitations

Despite its broad validation and ease of integration into electronic health record systems (42), its utility across all hospitalized patients remains limited due to its lack of validity in specific populations, such as pregnant (16) and cancer patients (17). Additionally, the PPS has not been effectively validated in certain racial groups, such as the Chinese population (43). Furthermore, it may not be suitable for predicting VTE risk in patients with acute respiratory conditions (44). One study showed that the PPS did not improve clinical outcomes when restricted to patients admitted for 72 h or more, after excluding upper-extremity DVT as an outcome, and after including all-cause mortality in a composite outcome (45). These findings suggest that its lack of dynamic risk adjustment and limited generalizability necessitate further evaluation to enhance its predictive accuracy for VTE risk assessment.

### 3.3 The IMPROVE predictive and associative scores

#### 3.3.1 Development, validation, and scoring system

The IMPROVE model was derived from large cohorts of hospitalized medical patients and is designed to support VTE risk stratification in medical inpatient settings. It was developed from the multinational International Medical Prevention Registry on VTE (IMPROVE), which enrolled 15,156 acutely ill medical inpatients across 52 hospitals in 12 countries between 2002 and 2006. The IMPROVE RAM enables clinicians to quantify baseline and evolving thrombotic risk. The four-item IMPROVE-Predictive Score is calculated upon admission (previous VTE = 3 points, known thrombophilia = 3, age over 60 years = 1, active cancer =  1); scores of 0–1, 2–3, and ≥4 indicate low, intermediate, and high risk, respectively, with the high-risk group (Table 2) showing a 90-day VTE incidence of approximately 5.7% (18, 19, 46). Risk can then be adjusted in the hospital by adding three dynamic variables: lower-limb paralysis (2 points), immobilization for 7 days or more (1 point), and ICU/CCU admission (1 point), resulting in the seven-factor IMPROVE-Associative Score (range: 0–12) (Table 2) (19, 20). External validation by Cobben et al. confirmed that this combined seven-factor model demonstrated the best discriminative performance in their cohort (AUC 0.66, 95% CI: 0.56–0.75), supporting its clinical utility while highlighting the need for further refinement in various settings (47).

**Table 2:** The IMPROVE predictive score, the IMPROVE associative score, the IMPROVEDD score.

The IMPROVE predictive score		The IMPROVE associative score		The IMPROVEDD score	
Variables	Points	Variables	Points	Variables	Points
Previous VTE	3	Previous VTE	3	Previous VTE	3
Malignancy Treated or untreated within the previous 6 months	1	Current Malignancy	2	Current Malignancy	2
Thrombophilia	3	Thrombophilia^1^	2	Thrombophilia^1^	2
Age >60 yrs	1	Age >60 yrs	1	Age >60 yrs	1
		Immobilization for at least 7 days	1	Immobilization for at least 7 days	1
		ICU or CCU admission	1	ICU or CCU admission	1
		Paralysis of the lower extremities during hospitalization	2	Paralysis of the lower extremities during hospitalization	2
				D-Dimer >2X Upper Limit Normal	2
VTE Risk on admission		VTE Risk		VTE Risk	
Low: Observed VTE risk <1%		Low Risk = 0–1.5		Predicted VTE Risk at 42-day	
0–1		Moderate Risk = 2–3		Score 0 = 0.4%	
Moderate		High Risk = ≥4		Score 1 = 0.6%	
2–3				Score 2 = 1.0%	
High: Observed VTE risk 5.7%		The ASH guidelines recommend VTE prophylaxis for a score of ≥2.		Score 3 = 1.7%	
≥4				Score 4 = 2.9%	
				Score >5 = 7.2%	
		3-month VTE Risk		Predicted VTE Risk at 77 days	
		0 = 0.4%		Score 0 = 0.5%	
		1 = 0.6%		Score 1 = 0.7%	
		2 = 1.0%		Score 2 = 1.0%	
		3 = 1.7%		Score 3 = 1.4%	
		4 = 2.9%		Score 4 = 1.9%	
		≥5 = 7.2%		Score >5 = 2.7%	

Clinical variables included in the IMPROVE predictive and associative models and the D-dimer–augmented IMPROVEDD variant for hospitalized medical patients. Adapted from the original IMPROVE model derivation and validation studies and the IMPROVEDD biomarker-augmented analysis (18–22, 46). This table summarizes model structure and does not represent new model derivation.

#### 3.3.2 Clinical implications and limitations

The IMPROVE RAM was derived from a large, geographically diverse registry of acutely ill medical patients and has since been embedded in several electronic health record (EHR) order sets. However, it's essential to note that the IMPROVE RAM was not designed to assess how thromboprophylaxis after admission affects the occurrence of VTE events, nor to determine how VTE prevention might influence the model's results, as patients were not randomly assigned to receive prophylaxis. Another limitation could be the reliance on clinical endpoints rather than ongoing monitoring of VTE, which may have led to an underestimation of VTE cases (18). Additionally, the method doesn’t account for new risk factors or biomarkers, such as D-dimer, leukocyte, or platelet counts.

### 3.4 The IMPROVEDD score

#### 3.4.1 Development, validation, and scoring system

Gibson and colleagues evaluated the association between D-dimer levels above twice the upper limit of normal (ULN) and the IMPROVE score's ability to predict nonfatal pulmonary embolism, VTE-related death, or symptomatic DVT in a cohort of 7,441 hospitalized, medically ill patients from the APEX trial. When D-dimer exceeded 2x ULN, an extra 2 points were added to the IMPROVE score, creating the IMPROVEDD score **(**Table 2). Baseline D-dimer levels were independently linked to symptomatic VTE over 77 days (21). In hospitalized patients with medical conditions, the modified IMPROVE VTE risk score, which includes high D-dimer levels as a biomarker, identified a group at nearly three times higher risk for VTE. For these patients, extended thromboprophylaxis with rivaroxaban for up to 35 days provides a notable benefit (22).

#### 3.4.2 Clinical implications and limitations

Gibson et al. emphasize that the IMPROVE clinical variables, combined with a D-dimer level at least twice the ULN, serve as independent predictors of in-hospital VTE. Since this evidence is based solely on the APEX trial, in which all participants received either standard enoxaparin or extended betrixaban, its relevance to settings with different or no prophylactic treatments is uncertain (21, 22). Importantly, elevated D-dimer levels may reflect inflammation, malignancy, or acute illness rather than thrombosis alone and should therefore be interpreted only within a validated multivariable framework.

### 3.5 The Geneva RAMs

#### 3.5.1 Development, validation, and scoring system

The Geneva score is a diagnostic risk-assessment model designed to estimate the clinical probability of pulmonary embolism in patients with suspected VTE, primarily in emergency and outpatient settings. Wicki et al. first introduced the Geneva score in 2001 within a single-center Swiss cohort of 1,090 patients from the emergency department (28). The 0- to 16-point scale assigns weighted values to eight objective variables: age ≥ 60 years, surgery within the past 4 weeks, prior DVT/PE, heart rate > 100 bpm, PaO₂ < 82 mmHg, PaCO₂ < 39 mmHg, and chest radiograph evidence of plate-like atelectasis or hemidiaphragm elevation. The resulting totals categorize clinical pre-test probability as low (0–4), intermediate (5–8), or high (>8) (Table 3). In 2006, Le Gal and colleagues simplified the score by removing chest radiography and arterial blood-gas measurements, resulting in the revised Geneva score (48). This version relies on eight easily accessible clinical variables: heart rate ≥ 75 bpm, age > 65 years, unilateral lower-limb pain with tenderness or edema, hemoptysis, prior DVT/PE, recent surgery or lower-limb fracture (<4 weeks), and active cancer, stratifying pulmonary embolism probability as low (0–3), intermediate (4–10), or high (≥11) points (Table 3). Later, in 2008, Klok et al. introduced the simplified revised Geneva score, which uses the same eight clinical variables but assigns one point to each (heart rate ≥ 95 bpm receives two points). Scores of 0–1, 2–4, and 5–7 indicate low (approximately 8%), intermediate (approximately 29%), and high (approximately 64%) pre-test probabilities, respectively. A binary cutoff of ≤2 vs. ≥3 (“PE unlikely” vs. “PE likely”) supports D-dimer–guided exclusion strategies (Table 3) (29). External validation has confirmed the model's strong discrimination and calibration; however, clinical use remains inconsistent, partly due to alert fatigue and the earlier, more cumbersome versions of the Geneva score, which may hinder the implementation of decision-support systems (30).

**Table 3:** Geneva scores.

The original GENEVA risk prediction score		The revised GENEVA prediction score		The simplified GENEVA risk prediction score	
Age		Age >65 years	1	Age >65 years	1
60–79 yrs	1	Previous DVT and or PE	3	Previous VTE event	1
80+ yrs	2	Surgery [under GA] or Lower Limb fracture in the previous 4 weeks	2	Previous surgery or fracture within 4 weeks	1
Previous VTE event		Active Malignancy [solid or hematological—currently active or considered cured within 12 months]	2	Active malignancy	1
Yes	2	Unilateral lower limb pain	3	Unilateral lower limb pain	1
No	0	Hemoptysis	2	Hemoptysis	1
Previous surgery within 4 weeks		Heart Rate		Heart Rate	
Yes	3	75–94 bpm	3	75–94 bpm	1
No	0	≥95 bpm	5	≥94 bpm	2
Heart rate >100 bpm		Pain in the lower limb, deep venous palpation, and unilateral edema	4	Pain on deep palpation of the lower limb and unilateral edema	1
Yes	1				
No	0				
PaCO_2_ [Arterial blood]					
<35 mmHg	2				
35–39 mmHg	1				
PaO_2_ [Arterial blood]					
<49 mmHg	4				
49–59 mmHg	3				
60–71 mmHg	2				
72–82 mmHg	1				
CXR					
Band atelectasis Elevated	1				
hemidiaphragm	1				
Interpretation					
• <5 Points—low probability of PE • 5–8 Points—moderate probability of PE • >8 Points—high probability of PE		• Low clinical probability for PE: 0–3 • Intermediate clinical probability for PE: 4–10 D-dimer testing: – Negative: Consider stopping further investigation – Positive: Imaging indicated • High clinical probability for PE > 60% probability of PE: ≥11 Imaging indicated		• <2 Points—low probability of PE • 2–4 Points—moderate probability of PE • 5–7 Points—high probability of PE	

Variables and point allocation of the original and revised Geneva scores used to estimate the pretest probability of pulmonary embolism in patients with suspected VTE. Adapted from the original Geneva score derivation and subsequent revisions and validation studies (28, 29, 48).

#### 3.5.2 Clinical implications and limitations

The Geneva RAMs streamline pre-test assessments and can be combined with D-dimer testing to safely exclude PE without imaging. Prospective validation in the ADJUST-PE management trial confirmed that the simplified RAM maintained safety and accuracy in 1,621 outpatients (30). However, both revised and simplified versions, originating from emergency ward cohorts, show reduced discrimination in general medical inpatients (AUC ≈ 0.58 and positive predictive value ≈ 3% in a 2024 head-to-head comparison of risk models) (31), limiting their usefulness for hospital-acquired VTE surveillance or thromboprophylaxis decisions. Their use in ward-based or perioperative populations should await validation specific to those groups.

### 3.6 Well scores

#### 3.6.1 Development, validation, and scoring system

The Wells score was developed to estimate the pretest probability of suspected VTE and is intended for diagnostic decision-making rather than routine thromboprophylaxis risk assessment. It was first proposed in 1995 and refined in 1997, which translates bedside findings into a quantitative pre-test probability (12). The updated model assigns +1 point for each of nine predictors: active cancer, paralysis/paresis or recent plaster immobilization of a lower limb, recent bed-rest > 3 days or major surgery within the previous 12 weeks, localized tenderness along the deep-venous system, entire leg swelling, calf circumference ≥ 3 cm larger than the asymptomatic leg, unilateral pitting oedema, collateral superficial (non-varicose) veins, and previous DVT, while an alternative diagnosis at least as likely as DVT carries −2 points. Totals stratify patients into low (≤0), intermediate (1–2), and high (≥3) probability groups, corresponding to observed proximal DVT prevalences of roughly 5%, 17%, and 53%, respectively (Table 4) (12, 49). The original well model was revised in 2003 to become the modified Wells. Three modifications were made: Twelve weeks were added to the recovery period following major surgery, for previously confirmed DVT, a tenth predictor was added and assigned one point, and the associated risk was divided into likely (≥2) categories and DVT unlikely (<2 points) (Table 4) (23). In 2008, Gibson et al. simplified the PE Wells rule by retaining the original seven predictors and assigning each a point, with a total score of ≤1 classifying PE as unlikely and a score > 1 as likely (Table 4). In their prospective cohort, the 3-month VTE rate after a negative D-dimer was nearly the same as that of the conventional and simplified rules (0.3% vs. 0.5%), confirming non-inferiority (24). A later single-center series by Al-Khafaji et al. showed that the Wells framework maintained excellent discrimination for DVT in both emergency department and ward patients (AUC ≈ 0.86) (25). Finally, Hendriksen and colleagues compared the original, modified, and simplified Wells rules with the revised and simplified Geneva scores in 598 outpatients. Failure rates after a normal D-dimer were lower with the Wells variants (1.2%–1.5%) than with either of the Geneva algorithms (2.7%–3.1%) (26).

**Table 4:** The wells PE score.

Criteria	Original	modified	Simplified
An alternative diagnosis is less likely than PE	3	2	1
Clinical signs and symptoms of a DVT	3	2	1
Immobilization for >3 days or surgery in the previous 4 weeks	1.5	1	1
Heart Rate > 100 bpm	1.5	1	1
Previous DVT-PE	1.5	1	1
Active cancer [on treatment or treatment in previous 6 months or palliative]	1	1	1
Hemoptysis	1	1	1
Cut-off for PE unlikely	≤4	≤2	≤1
**NOTE**: The original WELLS score [1998] employed a seven-component clinical prediction rule for PE. • Score <2: Low risk for PE • Score 2–6: Intermediate risk for PE • Score of >6: High risk for PE			
In 2000 the WELLS score for PE was revised to reduce the number of risk categories to two: Score >4: PE Likely • Score ≤ 4: PE Unlikely			

Criteria and point allocation for the Wells DVT and PE rules used to estimate pretest probability in patients with suspected VTE. Adapted from the original Wells score derivation studies and subsequent revisions (12, 23–26, 49).

#### 3.6.2 Clinical implications and limitations

In trauma patients, DVT can be reliably excluded with a Wells score of less than 1 (27). A modified Wells score, combined with a negative D-dimer test, can confidently exclude DVT in outpatients or individuals in primary care settings, as indicated by Geersing et al. (50). However, the Wells score exhibits limited effectiveness in patients with distal DVT, older patients, and those with a history of prior DVT (51–53).

### 3.7 Kucher RAM

#### 3.7.1 Development, validation, and scoring system

The Kucher RAM was created as a real-time, electronic alert embedded in the EHR to flag medical inpatients at an elevated risk of VTE (13). “Kucher alert” improved the prescribing of prophylactic anticoagulation and reduced 90-day symptomatic VTE by ∼approximately 40% (13). Sustained benefit was later confirmed in an observational follow-up, with a halving of VTE incidence when the alert system remained active (54). The RAM assigns weighted points to eight variables: prior VTE, active cancer, known hypercoagulability (3 points each); recent major surgery (2 points); and age > 75 years, body-mass index > 29 kg m², bed rest ≥ 3 days, or estrogen/hormone therapy (1 point each). A cumulative score of 4 or higher triggers a “Kucher alert”, prompting clinicians to initiate pharmacological or mechanical prophylaxis; lower totals stratify patients into intermediate- and low-risk categories.

#### 3.7.2 Clinical implications and limitations

Subsequent external validation in a 20-hospital U.S. cohort showed that the Kucher RAM had the weakest discrimination among five competing scores (c-statistic approximately 0.55). This raises concerns about its generalizability outside the environment in which it was developed. The study also found that most alerts were ignored, a pattern attributed to alert fatigue caused by the indiscriminate firing of alerts for all medical inpatients (55). Current guidance now recommends limiting e-alerts to truly high-risk patients and pairing them with a parallel bleed-risk prompt, an element missing from the original design (56).

## 4 Comparative evaluations of VTE RAM

Because VTE RAMs differ substantially in their derivation populations and intended clinical use, comparative interpretation should be guided by scenario-specific validation and awareness of population-specific limitations. Comparative evidence indicates that the discriminatory performance of VTE RAMs varies significantly across contexts. This variability is illustrated in Figure 1, which outlines the evolution of both established and emerging approaches to VTE risk assessment. In mixed medical-ward cohorts, the Caprini score has outperformed the Padua score, demonstrating better sensitivity, specificity, and overall accuracy. In surgical series, its prognostic usefulness is generally similar to that observed in the original derivation studies (38). For bedside diagnosis of suspected lower-limb thrombosis, the Wells DVT rule remains the most dependable, surpassing both the Caprini and Padua rules. Assessments of PE in adults aged 65 and older in ambulatory settings show comparable accuracy for the Wells and revised Geneva rules (57).

Electronic decision-support introduces an additional layer: the simplified four-factor model developed by Woller et al. (which includes previous VTE, cancer, immobility ≥ 3 days, and central venous line) demonstrates better 90-day predictive accuracy on medical wards compared to the original eight-factor Kucher alert (20). A systematic review of 18 validation studies indicates the most substantial evidence for the Caprini score in surgical groups and the Padua or IMPROVE scores in acutely ill medical inpatients. However, it also notes the scarcity of consistent, direct comparisons in homogeneous populations (58). Overall, these findings emphasize the importance of selecting models based on the clinical context: Caprini for diverse surgical or general inpatients, Padua or IMPROVE for non-surgical medical wards, Geneva derivatives for outpatient PE triage, and custom electronic algorithms where robust decision-support systems are available. A summary of RAM features and suitable clinical settings is provided in Table 5.

**Table 5:** Comparison of VTE risk assessment tools.

Tool	Sensitivity	Specificity	Predictive values	Clinical utility and ease of use	Strengths	Weaknesses	Best-validated population
Caprini Score	High for surgical patients, particularly in identifying those at high risk of VTE	Moderate, as it may overestimate risk in some low-risk patients	High PPV for identifying surgical patients likely to benefit from prophylaxis; NPV moderate in non-surgical settings	Easy to use, widely validated	Tailored for surgical patients; comprehensive; identifies high-risk individuals effectively	Limited utility outside of surgical populations; scoring may vary depending on clinical interpretation	Surgical patients
Padua Score	High for identifying medical patients at risk of VTE during hospitalization	Moderate, with potential false positives in patients with borderline scores	High PPV for hospitalized patients who meet criteria for prophylaxis; NPV moderate for patients not requiring intervention	Simple checklist approach	Specific to hospitalized medical patients; validated for non-surgical populations	Less useful in mixed or surgical settings; may not capture all risk factors	Medical inpatients
Wells Score	Moderate for detecting symptomatic VTE, varies based on cutoffs	High for ruling out VTE in low-probability patients, especially with D-dimer testing	High PPV for symptomatic DVT/PE in high-probability patients; NPV high when combined with D-dimer testing	Straightforward with clinical judgment	Effective for symptomatic DVT/PE; stratifies patients into categories for diagnostic decisions	Requires clinical judgment, which may introduce variability; less applicable for asymptomatic patients	Ambulatory, outpatient, or emergency department patients with suspected VTE
Kucher Score	Moderate, particularly in ICU populations where the tool is most applicable	Moderate, as it may not capture all at-risk ICU patients	Moderate PPV for predicting VTE in critically ill patients; NPV also moderate in the absence of other significant risk factors	Focused on critically ill patients	Useful in ICU settings; identifies at-risk patients who may benefit from thromboprophylaxis	Limited validation outside ICU; may underestimate risk in non-ICU patients	Medical inpatients
IMPROVE Score	Moderate for hospitalized medical patients, particularly those with multiple risk factors	High, as it incorporates both VTE and bleeding risk, leading to more targeted assessments	Moderate PPV due to its focus on concurrent VTE and bleeding risk; NPV higher for patients with low combined risk scores	Slightly more complex than others as it incorporates multiple variables that assess the risk of VTE	Validated for hospitalized medical patients; incorporates bleeding risk alongside thrombosis risk	More complex scoring system; potential for lower applicability outside medical patients	Medical inpatients

VTE, venous thromboembolism; DVT, deep vein thrombosis; PE, pulmonary embolism; PPV, positive predictive value—the likelihood that patients identified as high-risk truly develop VTE; NPV, negative predictive value—the likelihood that patients identified as low-risk truly do not develop VTE.

For sensitivity, specificity, and predictive values, High: ≥90% Moderate: 70%–89%, Low: <70%.

Comparison of derivation populations intended clinical use, strengths, limitations, and best-validated populations across established VTE RAMs. Synthesized from original model publications, external validation studies, and clinical guideline recommendations (10–15, 38–40, 57–61).This table represents a narrative comparison rather than a pooled quantitative analysis.

## 5 VTE RAM in special populations

### 5.1 Cancer patients

VTE remains one of the most consequential complications of malignancy, occurring five- to nine-fold more frequently than in the general population and affecting roughly 13% of patients within 12 months of starting chemotherapy (58, 60). This excess risk reflects a convergence of tumor-related factors (site, stage, histology), treatment exposures (cytotoxic or anti-angiogenic agents, central venous catheters, recent surgery), and patient characteristics such as advanced age, immobility, obesity, and prior VTE (61, 62). Robust RAMs are therefore indispensable for guiding prophylaxis. The Khorana score, the most extensively validated oncology RAM, assigns points for tumor site, thrombocytosis, anemia, leukocytosis, and elevated body mass index; current guidelines define a score ≥ 2 as “intermediate-high risk” (Table 6), a threshold adopted after the AVERT and CASSINI trials demonstrated prophylactic benefit in this group (63, 64). Its evidence base, however, is derived almost entirely from ambulatory cohorts receiving chemotherapy, leaving its calibration for inpatients uncertain.

**Table 6:** Khorana score for prediction of VTE in cancer patients.

Variable	Points
Site of cancer^a^	
○ Very high-risk cancer (stomach, pancreas)	2
○ High-risk cancer (lung, lymphoma, gynecological, bladder, or testicular)	1
2. Prechemotherapy platelet count ≥350 × 10^9^/L	1
3. Prechemotherapy hemoglobin level <100 g/L or use of red cell growth factors	1
4. Prechemotherapy leukocyte count >11 × 109/L	1
5. Body mass index ≥35 kg/m^2^	1
Risk categories	
Intermediate or high risk for VTE	≥2
Low risk	<2

Variables and point allocation of the Khorana score used to estimate venous thromboembolism risk in ambulatory patients with cancer initiating systemic therapy. Adapted from the original Khorana score derivation study and subsequent validation and guideline summaries (63, 64).

a The AVERT trial included brain tumors as very high-risk cancer and myeloma as high-risk cancer (63).

Several alternative RAMs (PROTECHT, CONKO, ONKOTEV, TiC-Onco, COMPASS-CAT) incorporate treatment-specific or biomarker variables, yet remain incompletely validated, particularly in hospitalized populations where immobility, perioperative status, and disease progression compound thrombotic risk (65–69). Current NCCN guidance accordingly recommends routine pharmacological prophylaxis for nearly all hospitalized oncology patients, emphasizing the need for individualized assessment in the absence of an inpatient-specific tool (62). Preliminary data suggest that a Khorana score of ≥2 retains some prognostic value in inpatient settings. However, it fails to capture the dynamic clinical and laboratory changes that characterize the inpatient course (70). General medical RAMs, such as Padua, IMPROVE, or Geneva, include “active cancer” as a binary variable, yet they omit cancer-specific nuances, which limits their discriminative capacity in this setting (11, 21, 46, 48). Accordingly, the development and prospective validation of an oncology-specific inpatient RAM that potentially integrates real-time biomarkers, performance status, and treatment details represents critical priorities to refine risk stratification, optimize prophylaxis strategies, and ultimately improve cancer-related outcomes. Although several oncology-specific RAMs exist, including the Khorana score and its derivatives, most were developed and validated exclusively in ambulatory cancer populations. Hospitalized oncology patients experience dynamic changes in mobility, inflammatory burden, infection risk, treatment toxicity, and disease severity that are not captured by outpatient-derived models. As a result, extrapolation of these scores to inpatient settings may lead to misclassification of thrombotic risk. Population-specific recalibration and prospective validation of VTE RAMs tailored to hospitalized cancer patients are therefore needed, potentially incorporating dynamic clinical variables, biomarkers, and treatment-related factors.

### 5.2 Pregnant and the puerperium patients

VTE is a leading cause of maternal morbidity and mortality, responsible for 1.4–4.6 deaths per 100,000 live births, and its incidence is five to nine times higher during pregnancy than in the non-pregnant population (71). The risk peaks during the first six weeks postpartum, when rapid hemostatic rebound, endothelial injury from delivery, and lingering immobility combine with venous stasis caused by the gravid uterus. Additional factors such as advanced age, obesity, inherited or acquired thrombophilia, malignancy, prolonged bed rest, and prior VTE further increase the risk, but precise clinical stratification remains challenging (72). Several RAMs aim to quantify this risk. The Royal College of Obstetricians and Gynecologists (RCOG) and the Swedish Society of Obstetrics & Gynecology (SFOG) use cumulative point systems that trigger low-molecular-weight heparin prophylaxis at predefined thresholds (73–75). In contrast, the American College of Obstetricians & Gynecologists (ACOG) depends on clinician judgment without formal scoring (76). Specialized tools include the dynamic Lyon score, which combines laboratory markers, and obstetric versions of the Caprini and Padua scores that account for factors like pre-eclampsia, C-section, and postpartum immobility (41, 77, 78). Validation data are inconsistent. A Chinese case-control cohort reported good discrimination for the RCOG model (AUC 0.83) (79); however, UK researchers found that adding biomarkers, including D-dimer, LDL, and white blood cell count, significantly improved its accuracy (75). In a 6,094-delivery series, RCOG and Caprini classified 62% and 94% of women as high-risk, respectively, whereas Padua flagged fewer than 1%. None of the tools identified all postpartum events (41). A systematic review of 19 RAMs confirmed sensitivities and specificities ranging from 0% to 100%, indicating poor calibration and limited generalizability (73). The most recent prospective comparison showed that the modified Caprini and Swedish scores demonstrated the best overall discrimination (AUC approximately 0.80), although each sacrificed either sensitivity or specificity (80). Since no RAM has been prospectively validated for hospitalized obstetric patients, current guidelines recommend near-universal LMWH prophylaxis during obstetric admissions, while highlighting the importance of personalized assessment (74, 76). Improving existing models or creating new, inpatient-specific tools that include real-time biomarkers, mobility measures, and treatment details remains an essential research focus to optimize prophylaxis and decrease pregnancy-related VTE. Despite the availability of several obstetric-specific RAMs, none have undergone robust prospective validation in hospitalized pregnant or postpartum populations. Pregnancy-specific physiological changes, including progressive hypercoagulability, venous stasis, and abrupt postpartum hemostatic shifts, are incompletely captured by existing models. Consequently, current RAMs may either overestimate or underestimate VTE risk in this population. These limitations highlight the need for population-specific recalibration and prospective validation of VTE RAMs designed specifically for hospitalized obstetric patients.

### 5.3 Pediatric patients

No widely accepted or prospectively validated VTE RAM currently exists for hospitalized pediatric patients. Pediatric VTE prevention strategies are primarily guided by expert consensus recommendations and identification of individual risk factors rather than formalized scoring systems (80, 81). Although clinical practice guidelines describe pediatric-specific thrombotic risk factors, these do not constitute validated RAM (80).

A pediatric hospital-acquired thrombosis risk model has been proposed by the Children's Hospital-Acquired Thrombosis Consortium (82); however, this model remains limited to derivation and early validation cohorts and has not yet been externally validated or incorporated into major clinical guidelines (80). Consequently, pediatric VTE risk assessment in routine practice continues to rely primarily on institutional protocols and clinical judgment. Adult-derived RAMs, including Caprini, Padua, and IMPROVE, are not directly applicable to pediatric populations due to age-dependent differences in hemostatic physiology and provoking risk factors, underscoring a persistent evidence gap (81).

### 5.4 Geriatrics patients

Although advanced age is incorporated as a risk variable in several adult VTE risk-assessment models, no VTE RAM has, to date, explicitly been derived or prospectively validated for geriatric inpatients. Older adults present unique challenges due to frailty, multimorbidity, reduced mobility, sarcopenia, renal dysfunction, and polypharmacy, all of which influence both thrombotic and bleeding risk but are not adequately captured by existing models (83).

Evidence from diagnostic studies indicates that the performance of commonly used clinical prediction rules and biomarkers declines with advancing age. In particular, the specificity of D-dimer testing decreases substantially in older adults, leading to higher false-positive rates, although age-adjusted cutoffs improve diagnostic efficiency without constituting a prophylactic risk-assessment model (52). Consequently, VTE risk stratification in geriatric inpatients continues to rely on imperfect extrapolation of adult-derived RAMs combined with clinical judgment. These limitations underscore the need for geriatric-specific recalibration or derivation studies that incorporate functional status, frailty, and competing bleeding risks (83, 84).

Overall, currently available VTE RAMs were developed mainly in general adult populations and do not perform uniformly across all inpatient subgroups. In patients with malignancy, pregnancy and the puerperium, pediatric populations, and older adults, distinct physiological features, evolving clinical risk profiles, and competing bleeding risks reduce the accuracy of existing tools. These gaps highlight the need for population-specific recalibration and prospective validation to support individualized thromboprophylaxis in these settings better. Population-specific recalibration and prospective validation, particularly in inpatient settings, are essential.

## 6 Emerging biomarkers, genomic insights, and AI-Driven tools for VTE risk prediction

Novel biomarkers and data science approaches are being explored to address the poor specificity of D-dimer and to shift VTE prediction from a “rule-out” approach to accurate risk stratification. Soluble P-selectin (sP-selectin), released by activated platelets and endothelial cells, consistently indicates a thrombogenic state; plasma levels predict both initial and recurrent events with discrimination similar to that of D-dimer, especially in cancer-related VTE (85, 86). Thrombin generation (TG) assays combine multiple pro- and anticoagulant influences. Elevated peak thrombin or endogenous thrombin potential (ETP) independently forecasts first VTE and recurrence, although variability between laboratories still limits clinical application. Conversely, common inflammatory markers (CRP, IL-6) lose predictive power when obesity and coexisting conditions are considered and are not viewed as standalone risk indicators (32).

Genomics is reshaping risk profiling. A 2023 genome-wide meta-analysis identified 93 loci—62 of which were previously unknown. It showed that a polygenic risk score (PRS) places individuals in the top 0.1% at a risk comparable to that of Factor V Leiden carriers, while those in the lowest decile approach the population baseline. Integrating PRS with clinical factors significantly improves discrimination and may limit unnecessary anticoagulation (33).

Machine-learning (ML) models further enhance prediction. In a young-to-middle-aged inpatient cohort, a support vector machine algorithm that incorporated routine laboratory tests (e.g., fibrinogen, prothrombin time) achieved 87% accuracy and an AUC of 0.94, surpassing that of logistic regression (34). A meta-analysis of 12 studies (>51,000 patients) confirmed high pooled sensitivity and specificity for AI-based tools. However, interpretability and prospective validation remain barriers to deployment (35). Clinician surveys reinforce these concerns, highlighting the importance of transparent algorithms and the need for ongoing bedside judgment (36).

Despite their promise, several significant barriers remain that hinder the translation of promising research into routine clinical practice. For example, integration into existing clinical workflows proves challenging: many healthcare institutions still rely on legacy systems, and AI tools may not smoothly interface with EHR, laboratory systems, or hospital information systems, a barrier commonly reported in implementation studies (37, 87).

Strong safeguards are required to protect data privacy, confidentiality, and informed consent, especially when AI relies on continuous monitoring and cloud-based systems (88, 89). Regulatory compliance (e.g., GDPR, HIPAA) and robust cybersecurity measures are essential to prevent breaches that could jeopardize patient safety (90, 91). Additionally, the “black-box” nature of many ML models raises concerns about transparency and accountability, hindering clinician trust and adoption. Therefore, ethical frameworks promoting explainability, governance, and professional oversight are necessary for safe bedside use. AI should complement, not replace, clinical judgment, and wider implementation will depend on secure integration and prospective validation (92–94).

## 7 Translating VTE-RAM into routine care: guidelines, barriers, and successful system solutions

The 2024 International Consensus on preventing VTE highlights VTE risk stratification and reviews validated RAMs for medical, surgical, pregnant, postpartum, and cancer patients (95). The 2018 ASH guideline recommends using the Padua and IMPROVE models for VTE risk and the IMPROVE model for bleeding risk, advocating for integrated assessments in clinical decision-making (7). In 2020, the American Heart Association called for a 20% reduction in hospital-acquired VTE by 2030, stressing the importance of risk assessments and reporting for hospitalized patients to enable benchmarking and pay-for-performance (96). The Joint Commission International (JCI) views VTE prevention as vital to patient safety, requiring hospitals to utilize measurement data to evaluate services for high-risk patients, incorporate findings into quality initiatives, enhance safety practices, and assess the effectiveness of actions within set timeframes (97).

Despite existing guidelines' recommendations and JCI endorsement, a significant gap remains in transitioning towards individualized risk assessment using RAM instead of standard, universal prophylaxis or under-prescribing (98). A 2019–2020 World Thrombosis Day survey of 213 hospitals in 34 countries found that although 84% of institutions reported using a RAM, only 68% made completion compulsory, and fewer than 9% of countries had a national mandate for routine assessment (99). Knowledge gaps contribute to the fact that almost 45% of clinicians in separate audits had never been trained to apply a RAM, and barely one-third based prophylaxis decisions on one (100). High clinical workload, documentation demands, and “click fatigue” further erode compliance, while cognitive bias can cause clinicians to override electronic prompts. Defensive practice driven by liability fears adds to inappropriate anticoagulant use, and variable local protocols create confusion across wards and institutions (101).

Several institution-level solutions have proven effective. England's mandatory electronic tool, linked to reimbursement, has prevented over 900 fatal postoperative PEs within two years (102). A University of Michigan study involving 223,405 inpatients (2008–2016) compared mandatory with voluntary Caprini risk assessments. Making assessments mandatory increased prophylaxis orders from 65% to 79%. The risk of VTE dropped by 14%, mainly due to a 36% reduction in in-hospital events. Reversing the mandate negated these gains, but re-establishing the requirement restored them (103). Additionally, in a before–after study at Boston University Medical Center involving patients undergoing general and vascular surgery, introducing the mandatory Caprini-score pathway reduced the incidence of DVT from 1.9% to 0.3%, representing an 84% relative decrease, and decreased the incidence of PE by 55% (104). Overall, integrating mandatory electronic risk scoring at admission and when clinical status changes, with targeted education, audit-feedback processes, and standardized protocols, offers a practical way to bridge the implementation gap and fully achieve appropriate thromboprophylaxis.

## 8 Conclusions and future directions

VTE continues to rank among the most preventable causes of in-hospital morbidity and mortality. Over the past three decades, a diverse portfolio of RAM, including Caprini, Padua, IMPROVE, Wells, Geneva, Kucher e-alerts, and more recent biomarker- or machine-learning-enhanced tools, has emerged to replace empiric, “one-size-fits-all” prophylaxis with risk-tailored strategies. When applied within their intended populations, these RAMs reduce both undertreatment and unnecessary anticoagulation, thereby improving clinical outcomes and resource utilization.

Despite progress, several issues remain. Performance stays context-dependent; the model's ability to discriminate often worsens when a RAM is used outside its original cohort. High-quality validation is especially limited in hospitalized oncology, obstetric, pediatric, and geriatric populations. Additionally, implementation data highlight system-level challenges, including inadequate clinician training, cognitive biases, documentation overload, alert fatigue, and variability in local protocols, all of which hinder adherence to guideline-based risk stratification. However, experience from national efforts in the UK and single-center programs using mandatory, workflow-integrated electronic RAMs shows these barriers can be addressed, leading to long-term reductions in VTE rates.

Future priorities should therefore include (i) rigorous external validation and recalibration of existing RAMs in underrepresented patient groups; (ii) incorporation of dynamic variables such as laboratory biomarkers, mobility metrics, and evolving treatment exposures into adaptable algorithms; and (iii) development of transparent, user-centered digital decision-support systems that seamlessly integrate thrombosis and bleeding risk, minimize cognitive load, and are supported by audit-feedback mechanisms and institutional incentives. Addressing these areas is crucial for translating methodological advances into consistent bedside practice and for achieving meaningful, system-wide reductions in hospital-associated VTE.

## References

[1] Centers for Disease Control and Prevention. Data and Statistics on Venous Thromboembolism (blood Clots). Atlanta (GA): CDC (2025). Available online at: https://www.cdc.gov/blood-clots/data-research/facts-stats/index.html (Accessed July 5, 2025).

[2] Clapham RE Roberts LN. A systematic approach to venous thromboembolism prevention: a focus on UK experience. Res Pract Thromb Haemost. (2022) 7(1):100030. 10.1016/j.rpth.2022.100030 36760776 PMC9903667

[3] Oleksiuk-Bójko M Lisowska A. Venous thromboembolism: why is it still a significant health problem? Adv Med Sci. (2023) 68(1):10–20. 10.1016/j.advms.2022.10.002 36368288

[4] Wendelboe AM Raskob GE. Global burden of thrombosis: epidemiologic aspects. Circ Res. (2016) 118(9):1340–7. 10.1161/CIRCRESAHA.115.306841 27126645

[5] Pastori D Cormaci VM Marucci S Franchino G Del Sole F Capozza A A comprehensive review of risk factors for venous thromboembolism: from epidemiology to pathophysiology. Int J Mol Sci. (2023) 24(4):3169. 10.3390/ijms24043169 36834580 PMC9964264

[6] Porterfield L Davis JW Weller SC Chen L Wilkinson G. Does hormone therapy exacerbate other venous thromboembolism risk factors? Menopause. (2024) 31(2):123–9. 10.1097/GME.0000000000002305 38270903

[7] Schünemann HJ Cushman M Burnett AE Kahn SR Beyer-Westendorf J Spencer FA American society of hematology 2018 guidelines for management of venous thromboembolism: prophylaxis for hospitalized and nonhospitalized medical patients. Blood Adv. (2018) 2(22):3198–225. 10.1182/bloodadvances.2018022954. *Blood Adv*. (2023) **7**(9):1671. doi: 10.1182/bloodadvances.2022008147. Erratum for: *Blood Adv*. (2018) **2**(22):3198–25. 30482763 PMC6258910

[8] Venous Thromboembolism in Over 16s: Reducing the Risk of Hospital-acquired deep Vein Thrombosis or Pulmonary Embolism. London: National Institute for Health and Care Excellence (NICE) (2019). PMID: 32924386. 32924386

[9] Qaseem A Chou R Humphrey LL Starkey M Shekelle P. Clinical guidelines committee of the American college of physicians. Venous thromboembolism prophylaxis in hospitalized patients: a clinical practice guideline from the American college of physicians. Ann Intern Med. (2011) 155(9):625–32. 10.7326/0003-4819-155-9-201111010-00011 22041951

[10] Caprini JA. Thrombosis risk assessment as a guide to quality patient care. Dis Mon. (2005) 51(2–3):70–8. 10.1016/j.disamonth.2005.02.003 15900257

[11] Barbar S Noventa F Rossetto V Ferrari A Brandolin B Perlati M A risk assessment model for the identification of hospitalized medical patients at risk for venous thromboembolism: the Padua prediction score. J Thromb Haemost. (2010) 8(11):2450–7. 10.1111/j.1538-7836.2010.04044.x 20738765

[12] Wells PS Anderson DR Bormanis J Guy F Mitchell M Gray L Value of assessment of pretest probability of deep-vein thrombosis in clinical management. Lancet. (1997) 350(9094):1795–8. 10.1016/S0140-6736(97)08140-3 9428249

[13] Kucher N Koo S Quiroz R Cooper JM Paterno MD Soukonnikov B Electronic alerts to prevent venous thromboembolism among hospitalized patients. N Engl J Med. (2005) 352(10):969–77. 10.1056/NEJMoa041533 15758007

[14] Pandor A Tonkins M Goodacre S Sworn K Clowes M Griffin XL Risk assessment models for venous thromboembolism in hospitalised adult patients: a systematic review. BMJ Open. (2021) 11(7):e045672. 10.1136/bmjopen-2020-045672 34326045 PMC8323381

[15] Cronin M Dengler N Krauss ES Segal A Wei N Daly M Completion of the updated caprini risk assessment model (2013 version). Clin Appl Thromb Hemost. (2019) 25:1076029619838052. 10.1177/1076029619838052 30939900 PMC6714938

[16] Caroline H Christine S Kathleen G Teresa W Kristin D Funminiyi T. Venous thromboembolism: padua prediction score in the obstetric patient [26J]. Obstet Gynecol. (2016) 127:88S. 10.1097/01.AOG.0000483795.00678.28

[17] Łukaszuk RF Dolna-Michno J Plens K Czyżewicz G Undas A. The comparison between Caprini and Padua VTE risk assessment models for hospitalised cancer patients undergoing chemotherapy at the tertiary oncology department in Poland: is pharmacological thromboprophylaxis overused? Contemp Oncol. (2018) 22(1):31–6. 10.5114/wo.2018.74391 PMC5909728 29692661

[18] Tapson VF Decousus H Pini M Chong BH Froehlich JB Monreal M Venous thromboembolism prophylaxis in acutely ill hospitalized medical patients: findings from the international medical prevention registry on venous thromboembolism. Chest. (2007) 132(3):936–45. 10.1378/chest.06-2993 17573514

[19] Rosenberg D Eichorn A Alarcon M McCullagh L McGinn T Spyropoulos AC. External validation of the risk assessment model of the international medical prevention registry on venous thromboembolism (IMPROVE) for medical patients in a tertiary health system. J Am Heart Assoc. (2014) 3(6):e001152. 10.1161/JAHA.114.001152 25404191 PMC4338701

[20] Woller SC Stevens SM Jones JP Lloyd JF Evans RS Aston VT Derivation and validation of a simple model to identify venous thromboembolism risk in medical patients. Am J Med. (2011) 124(10):947–954.e2. 10.1016/j.amjmed.2011.06.004. Erratum in: *Am J Med*. (2012) **125**(10):e27. 21962315

[21] Gibson CM Spyropoulos AC Cohen AT Hull RD Goldhaber SZ Yusen RD The IMPROVEDD VTE risk score: incorporation of D-dimer into the IMPROVE score to improve venous thromboembolism risk stratification. TH Open. (2017) 1(1):e56–65. 10.1055/s-0037-1603929 31249911 PMC6524839

[22] Spyropoulos AC Lipardi C Xu J Peluso C Spiro TE De Sanctis Y Modified IMPROVE VTE risk score and elevated D-dimer identify a high venous thromboembolism risk in acutely ill medical population for extended thromboprophylaxis. TH Open. (2020) 4(1):e59–65. 10.1055/s-0040-1705137 32190813 PMC7069762

[23] Wells PS Anderson DR Rodger M Forgie M Kearon C Dreyer J Evaluation of D-dimer in the diagnosis of suspected deep-vein thrombosis. N Engl J Med. (2003) 349(13):1227–35. 10.1056/NEJMoa023153 14507948

[24] Gibson NS Sohne M Kruip MJ Tick LW Gerdes VE Bossuyt PM Further validation and simplification of the wells clinical decision rule in pulmonary embolism. Thromb Haemost. (2008) 99(1):229–34. 10.1160/TH07-05-0321 18217159

[25] Khafaji MA Marghalani M Alghamdi I Alsulami I Asaad W. Diagnostic accuracy of well’s score in clinically suspected deep venous thrombosis. JKAU Med Sci. (2022) 29(2):25–34. 10.4197/Med.29-2.4

[26] Hendriksen JM Geersing GJ Lucassen WA Erkens PM Stoffers HE van Weert HC Diagnostic prediction models for suspected pulmonary embolism: systematic review and independent external validation in primary care. Br Med J. (2015) 351:h4438. 10.1136/bmj.h4438 26349907 PMC4561760

[27] Modi S Deisler R Gozel K Reicks P Irwin E Brunsvold M Wells criteria for DVT is a reliable clinical tool to assess the risk of deep venous thrombosis in trauma patients. World J Emerg Surg. (2016) 11:24. 10.1186/s13017-016-0078-1 27279896 PMC4898382

[28] Wicki J Perneger TV Junod AF Bounameaux H Perrier A. Assessing clinical probability of pulmonary embolism in the emergency ward: a simple score. Arch Intern Med. (2001) 161(1):92–7. 10.1001/archinte.161.1.92 11146703

[29] Klok FA Mos IC Nijkeuter M Righini M Perrier A Le Gal G Simplification of the revised Geneva score for assessing clinical probability of pulmonary embolism. Arch Intern Med. (2008) 168(19):2131–6. 10.1001/archinte.168.19.2131 18955643

[30] Robert-Ebadi H Mostaguir K Hovens MM Kare M Verschuren F Girard P Assessing clinical probability of pulmonary embolism: prospective validation of the simplified Geneva score. J Thromb Haemost. (2017) 15(9):1764–9. 10.1111/jth.13770 28688113

[31] Häfliger E Kopp B Darbellay Farhoumand P Choffat D Rossel JB Reny JL Risk assessment models for venous thromboembolism in medical inpatients. JAMA Netw Open. (2024) 7(5):e249980. 10.1001/jamanetworkopen.2024.9980 38728035 PMC11087835

[32] Salavati M Arabshomali A Nouranian S Shariat-Madar Z. Overview of venous thromboembolism and emerging therapeutic technologies based on nanocarriers-mediated drug delivery systems. Molecules. (2024) 29(20):4883. 10.3390/molecules29204883 39459251 PMC11510185

[33] Ghouse J Tragante V Ahlberg G Rand SA Jespersen JB Leinøe EB Genome-wide meta-analysis identifies 93 risk loci and enables risk prediction equivalent to monogenic forms of venous thromboembolism. Nat Genet. (2023) 55(3):399–409. 10.1038/s41588-022-01286-7 36658437

[34] Liu H Yuan H Wang Y Huang W Xue H Zhang X. Prediction of venous thromboembolism with machine learning techniques in young-middle-aged inpatients. Sci Rep. (2021) 11(1):12868. 10.1038/s41598-021-92287-9 34145330 PMC8213829

[35] Wang Q Yuan L Ding X Zhou Z. Prediction and diagnosis of venous thromboembolism using artificial intelligence approaches: a systematic review and meta-analysis. Clin Appl Thromb Hemost. (2021) 27:10760296211021162. 10.1177/10760296211021162 34184560 PMC8246532

[36] Lam BD Dodge LE Zerbey S Robertson W Rosovsky RP Lake L The potential use of artificial intelligence for venous thromboembolism prophylaxis and management: clinician and healthcare informatician perspectives. Sci Rep. (2024) 14(1):12010. 10.1038/s41598-024-62535-9 38796561 PMC11127994

[37] Topol EJ. High-performance medicine: the convergence of human and artificial intelligence. Nat Med. (2019) 25(1):44–56. 10.1038/s41591-018-0300-7 30617339

[38] Trabulsi N Khafagy AM Alhazmi LS Alghamdi AM Alzahrani AA Banaamah MM Caprini versus Padua venous thromboembolism risk assessment scores: a comparative study in hospitalized patients at a tertiary center. Saudi Med J. (2024) 45(4):362–8. 10.15537/smj.2024.45.4.20230954 38657986 PMC11147578

[39] Vardi M Ghanem-Zoubi NO Zidan R Yurin V Bitterman H. Venous thromboembolism and the utility of the Padua prediction score in patients with sepsis admitted to internal medicine departments. J Thromb Haemost. (2013) 11(3):467–73. 10.1111/jth.12108 23279085

[40] Zhang J Xie Y Yang L Yang M Xu R Liu D. Validation of risk assessment scores in predicting venous thromboembolism in patients with lung cancer receiving immune checkpoint inhibitors. BMC Pulm Med. (2024) 24(1):507. 10.1186/s12890-024-03323-z 39390440 PMC11468413

[41] Tran JP Stribling SS Ibezim UC Omere C McEnery KA Pacheco LD Performance of risk assessment models for peripartum thromboprophylaxis. Reprod Sci. (2019) 26(9):1243–8. 10.1177/1933719118813197 30486735

[42] Elias P Khanna R Dudley A Davies J Jacolbia R McArthur K Automating venous thromboembolism risk calculation using electronic health record data upon hospital admission: the automated Padua prediction score. J Hosp Med. (2017) 12(4):231–7. 10.12788/jhm.2714 28411291

[43] Peng Q Chen X Han Y Tang G Liu J Liu Y Applicability of the Padua scale for Chinese rheumatic in-patients with venous thromboembolism. PLoS One. (2022) 17(12):e0278157. 10.1371/journal.pone.0278157 36525417 PMC9757592

[44] Yang S Zhang Y Jiao X Liu J Wang W Kuang T Padua Prediction score may be inappropriate for VTE risk assessment in hospitalized patients with acute respiratory conditions: a Chinese single-center cohort study. Int J Cardiol Heart Vasc. (2023) 49:101301. 10.1016/j.ijcha.2023.101301 PMID: 38035260; PMCID: PMC10684791. 38035260 PMC10684791

[45] Hayssen H Sahoo S Nguyen P Mayorga-Carlin M Siddiqui T Englum B Ability of Caprini and Padua Risk-Assessment Models to Predict Venous Thromboembolism in a Nationwide Study. medRxiv [Preprint]. (2023). 2023.03.20.23287506. 10.1101/2023.03.20.23287506. Update in: *J Vasc Surg Venous Lymphat Disord*. (2024) **12**(2):101693. doi: 10.1016/j.jvsv.2023.101693. PMC11017967 37499868

[46] Spyropoulos AC Jr AF FitzGerald G Decousus H Pini M Chong BH Predictive and associative models to identify hospitalized medical patients at risk for VTE. Chest. (2011) 140(3):706–14. 10.1378/chest.10-1944 21436241

[47] Cobben MRR Nemeth B Lijfering WM Cannegieter SC. Validation of risk-assessment models for venous thrombosis in hospitalized medical patients. Res Pract Thromb Haemost. (2019) 3:1–9. 10.1002/rth2.12181

[48] Le Gal G Righini M Roy PM Sanchez O Aujesky D Bounameaux H Prediction of pulmonary embolism in the emergency department: the revised Geneva score. Ann Intern Med. (2006) 144(3):165–71. 10.7326/0003-4819-144-3-200602070-00004 16461960

[49] Segal JB Eng J Tamariz LJ Bass EB. Review of the evidence on diagnosis of deep venous thrombosis and pulmonary embolism. Ann Fam Med. (2007) 5(1):63–73. 10.1370/afm.648 17261866 PMC1783914

[50] Geersing GJ Zuithoff NP Kearon C Anderson DR Ten Cate-Hoek AJ Elf JL Exclusion of deep vein thrombosis using the wells rule in clinically important subgroups: individual patient data meta-analysis. Br Med J. (2014) 348:g1340. 10.1136/bmj.g1340 24615063 PMC3948465

[51] Sartori M Cosmi B Legnani C Favaretto E Valdré L Guazzaloca G The wells rule and D-dimer for the diagnosis of isolated distal deep vein thrombosis. J Thromb Haemost. (2012) 10(11):2264–9. 10.1111/j.1538-7836.2012.04895.x 22906051

[52] Schouten HJ Geersing GJ Koek HL Zuithoff NP Janssen KJ Douma RA Diagnostic accuracy of conventional or age adjusted D-dimer cut-off values in older patients with suspected venous thromboembolism: systematic review and meta-analysis. Br Med J. (2013) 346:f2492. 10.1136/bmj.f2492 23645857 PMC3643284

[53] van Dam LF Gautam G Dronkers CEA Ghanima W Gleditsch J von Heijne A Safety of using the combination of the wells rule and D-dimer test for excluding acute recurrent ipsilateral deep vein thrombosis. J Thromb Haemost. (2020) 18(9):2341–8. 10.1111/jth.14986 32613731 PMC7497055

[54] Beeler PE Kucher N Blaser J. Sustained impact of electronic alerts on rate of prophylaxis against venous thromboembolism. Thromb Haemost. (2011) 106(4):734–8. 10.1160/TH11-04-0220 21800010

[55] Greene MT Spyropoulos AC Chopra V Grant PJ Kaatz S Bernstein SJ Validation of risk assessment models of venous thromboembolism in hospitalized medical patients. Am J Med. (2016) 129(9):1001.e9–e18. 10.1016/j.amjmed.2016.03.031 27107925

[56] Maynard G. Preventing Hospital-Associated Venous Thromboembolism: A Guide for Effective Quality Improvement. 2nd ed. Rockville (MD): Agency for Healthcare Research and Quality (US) (2016). (AHRQ Publication No. 16-0001-EF).

[57] Gatot D Mardia AI. Differences of wells scores accuracy, caprini scores and Padua scores in deep vein thrombosis diagnosis. IOP Conf Ser Earth Environ Sci. (2018) 125:012131. 10.1088/1755-1315/125/1/012131

[58] Mulder FI Horváth-Puhó E van Es N van Laarhoven HWM Pedersen L Moik F Venous thromboembolism in cancer patients: a population-based cohort study. Blood. (2021) 137(14):1959–69. 10.1182/blood.2020007338 33171494

[59] Mull N Mitchell MD Umscheid CA Hecht TE. Clinical risk prediction scores for venous thromboembolism (VTE) in hospitalized medical and surgical patients: a systematic review [abstract]. J Hosp Med. (2017) 12(Suppl 2). Presented at: Hospital Medicine 2017; 2017 May 1–4; Las Vegas, NV, USA. Philadelphia, PA: Society of Hospital Medicine.

[60] Khorana AA Dalal M Lin J Connolly GC. Incidence and predictors of venous thromboembolism (VTE) among ambulatory high-risk cancer patients undergoing chemotherapy in the United States. Cancer. (2013) 119(3):648–55. 10.1002/cncr.27772 22893596

[61] Falanga A Ay C Di Nisio M Gerotziafas G Jara-Palomares L Langer F Electronic address: clinicalguidelines@esmo.org. Venous thromboembolism in cancer patients: eSMO clinical practice guideline. Ann Oncol. (2023) 34(5):452–67. 10.1016/j.annonc.2022.12.014 36638869

[62] National Comprehensive Cancer Network© (NCCN), NCCN Guidelines Version 2.2024 Cancer-Associated Venous Thromboembolic Disease Available online at: https://www.nccn.org/professionals/physician_gls/pdf/vte.pdf (Accessed September 20, 2025).

[63] Carrier M Abou-Nassar K Mallick R Tagalakis V Shivakumar S Schattner A Apixaban to prevent venous thromboembolism in patients with cancer. N Engl J Med. (2019) 380(8):711–9. 10.1056/NEJMoa1814468 30511879

[64] Khorana AA Soff GA Kakkar AK Vadhan-Raj S Riess H Wun T Rivaroxaban for thromboprophylaxis in high-risk ambulatory patients with cancer. N Engl J Med. (2019) 380(8):720–8. 10.1056/NEJMoa1814630 30786186

[65] Verso M Agnelli G Barni S Gasparini G LaBianca R. A modified Khorana risk assessment score for venous thromboembolism in cancer patients receiving chemotherapy: the protecht score. Intern Emerg Med. (2012) 7(3):291–2. 10.1007/s11739-012-0784-y 22547369

[66] Pelzer U Sinn M Stieler J Riess H. Primäre medikamentöse thromboembolieprophylaxe bei ambulanten patienten mit fortgeschrittenem pankreaskarzinom unter chemotherapie? [Primary pharmacological prevention of thromboembolic events in ambulatory patients with advanced pancreatic cancer treated with chemotherapy?]. Dtsch Med Wochenschr. (2013) 138(41):2084–8. (In German). 10.1055/s-0033-1349608 24085361

[67] Cella CA Di Minno G Carlomagno C Arcopinto M Cerbone AM Matano E Preventing venous thromboembolism in ambulatory cancer patients: the ONKOTEV study. Oncologist. (2017) 22(5):601–8. 10.1634/theoncologist.2016-0246 28424324 PMC5423517

[68] Muñoz Martín AJ Ortega I Font C Pachón V Castellón V Martínez-Marín V Multivariable clinical-genetic risk model for predicting venous thromboembolic events in patients with cancer. Br J Cancer. (2018) 118(8):1056–61. 10.1038/s41416-018-0027-8 29588512 PMC5931103

[69] Gerotziafas GT Taher A Abdel-Razeq H AboElnazar E Spyropoulos AC El Shemmari S A predictive score for thrombosis associated with breast, colorectal, lung, or ovarian cancer: the prospective COMPASS-cancer-associated thrombosis study. Oncologist. (2017) 22:1222–31. 10.1634/theoncologist.2016-0414 28550032 PMC5634762

[70] Patell R Rybicki L McCrae KR Khorana AA. Predicting risk of venous thromboembolism in hospitalized cancer patients: utility of a risk assessment tool. Am J Hematol. (2017) 92(6):501–7. 10.1002/ajh.24700 28240823 PMC5729904

[71] Farmakis IT Barco S Hobohm L Braekkan SK Connors JM Giannakoulas G Maternal mortality related to pulmonary embolism in the United States, 2003–2020. Am J Obstet Gynecol MFM. (2023) 5(1):100754. 10.1016/j.ajogmf.2022.100754 36155111

[72] Raia-Barjat T Edebiri O Chauleur C. Venous thromboembolism risk score and pregnancy. Front Cardiovasc Med. (2022) 9:863612. 10.3389/fcvm.2022.863612 35479289 PMC9037588

[73] Pandor A Daru J Hunt BJ Rooney G Hamilton J Clowes M Risk assessment models for venous thromboembolism in pregnancy and in the puerperium: a systematic review. BMJ Open. (2022) 12(10):e065892. 10.1136/bmjopen-2022-065892 36223963 PMC9562726

[74] Royal College of Obstetricians and Gynaecologists. Reducing the Risk of Venous Thromboembolism During Pregnancy and the Puerperium (Green-top Guideline 37a). London: RCOG (2015).

[75] Li H Wan S Pei J Zhang L Peng J Che R. Use of the RCOG risk assessment model and biomarkers to evaluate the risk of postpartum venous thromboembolism. Thromb J. (2023) 21(1):66. 10.1186/s12959-023-00510-6 37308997 PMC10259017

[76] American College of Obstetricians and Gynecologists’ Committee on Practice Bulletins—Obstetrics. ACOG Practice bulletin No. 196: thromboembolism in pregnancy. Obstet Gynecol. (2018) 132(1):e1–e17. 10.1097/AOG.0000000000002706 Erratum in: *Obstet Gynecol*. (2018) **132**(4):1068. doi: 10.1097/AOG.0000000000002923. 29939938

[77] Dargaud Y Rugeri L Fleury C Battie C Gaucherand P Huissoud C Personalized thromboprophylaxis using a risk score for the management of pregnancies with high risk of thrombosis: a prospective clinical study. J Thromb Haemost. (2017) 15(5):897–906. 10.1111/jth.13660 28231636

[78] Luo X Zhang W Zhou R Tu X Guo Q Yuan S Comparison of risk assessments for venous thromboembolism during the puerperium. Heliyon. (2023) 9(2):e13568. 10.1016/j.heliyon.2023.e13568 36846687 PMC9946853

[79] Ge YZ Zhang C Cai YQ Huang HF. Application of the RCOG risk assessment model for evaluating postpartum venous thromboembolism in Chinese women: a case-control study. Med Sci Monit. (2021) 27:e929904. 10.12659/MSM.929904 34230447 PMC8274362

[80] Monagle P Chan AKC Goldenberg NA Ichord RN Journeycake JM Nowak-Göttl U Antithrombotic therapy in neonates and children: antithrombotic therapy and prevention of thrombosis, 9th ed: American college of chest physicians evidence-based clinical practice guidelines. Chest. (2012) 141(2):e737S–801. 10.1378/chest.11-2308 Erratum in: *Chest*. (2014) **146**(6):1694. Dosage error in article text. Erratum in: *Chest*. (2014) **146**(5):1422. 22315277 PMC3278066

[81] Faustino EV Raffini LJ. Prevention of hospital-acquired venous thromboembolism in children: a review of published guidelines. Front Pediatr. (2017) 5:9. 10.3389/fped.2017.00009 28184368 PMC5266715

[82] Jaffray J Mahajerin A Branchford B Nguyen ATH Faustino EVS Silvey M A new risk assessment model for hospital-acquired venous thromboembolism in critically ill children: a report from the Children’s hospital-acquired thrombosis consortium. Pediatr Crit Care Med. (2022) 23(1):e1–9. 10.1097/PCC.0000000000002826 34406168 PMC8738123

[83] Clegg A Young J Iliffe S Rikkert MO Rockwood K. Frailty in elderly people. Lancet. (2013) 381(9868):752–62. 10.1016/S0140-6736(12)62167-9 23395245 PMC4098658

[84] Guyatt GH Akl EA Crowther M Gutterman DD Schünemann HJ, American College of Chest Physicians. Executive summary: antithrombotic therapy and prevention of thrombosis, 9th ed: american college of chest physicians evidence-based clinical practice guidelines. Chest. (2012) 141(2):7S–47. 10.1378/chest.1412S3 22315257 PMC3278060

[85] Shiwani MA Chico TJA Ciravegna F Mihaylova L. Continuous monitoring of health and mobility indicators in patients with cardiovascular disease: a review of recent technologies. Sensors. (2023) 23(12):5752. 10.3390/s23125752 37420916 PMC10300851

[86] Pabinger I Ay C. Biomarkers and venous thromboembolism. Arterioscler Thromb Vasc Biol. (2009) 29(3):332–6. 10.1161/ATVBAHA.108.182188 19228607

[87] Chomutare T Tejedor M Svenning TO Marco-Ruiz L Tayefi M Lind K Artificial intelligence implementation in healthcare: a theory-based scoping review of barriers and facilitators. Int J Environ Res Public Health. (2022) 19(23):16359. 10.3390/ijerph192316359 36498432 PMC9738234

[88] Alowais SA Alghamdi SS Alsuhebany N Alqahtani T Alshaya AI Almohareb SN Revolutionizing healthcare: the role of artificial intelligence in clinical practice. BMC Med Educ. (2023) 23:689. 10.1186/s12909-023-04698-z 37740191 PMC10517477

[89] Alanazi A. Assessing Clinicians’ legal concerns and the need for a regulatory framework for AI in healthcare: a mixed-methods study. Healthcare. (2025) 13(13):1487. 10.3390/healthcare13131487 40648512 PMC12249247

[90] Cohen IG Mello MM. HIPAA And protecting health information in the 21st century. JAMA. (2018) 320(3):231. 10.1001/jama.2018.5630 29800120

[91] Yuan B Li J. The policy effect of the general data protection regulation (GDPR) on the digital public health sector in the European union: an empirical investigation. Int J Environ Res Public Health. (2019) 16(6):1070. 10.3390/ijerph16061070 30934648 PMC6466053

[92] Subramanian M Wojtusciszyn A Favre L Boughorbel S Shan J Letaief KB Precision medicine in the era of artificial intelligence: implications in chronic disease management. J Transl Med. (2020) 18(1):472. 10.1186/s12967-020-02658-5 33298113 PMC7725219

[93] Gerke S Minssen T Cohen G. Ethical and legal challenges of artificial intelligence-driven healthcare. Artif Intell Healthc. (2020):295–336. 10.1016/b978-0-12-818438-7.00012-5

[94] Von Eschenbach WJ. Transparency and the black box problem: why we do not trust AI. Philos. Technol. (2021) 34:1607–22. 10.1007/s13347-021-00477-0

[95] Nicolaides AN Fareed J Spyropoulos AC Kakkar RHL Antignani PL Avgerinos E Prevention and management of venous thromboembolism. International consensus statement. Guidelines according to scientific evidence. Int Angiol. (2024) 43(1):1–222. 10.23736/S0392-9590.23.05177-5 38421381

[96] Henke PK Kahn SR Pannucci CJ Secemksy EA Evans NS Khorana AA American Heart Association Advocacy coordinating committee. Call to action to prevent venous thromboembolism in hospitalized patients: a policy statement from the American Heart Association. Circulation. (2020) 141(24):e914–31. 10.1161/CIR.0000000000000769. Erratum in: *Circulation*. (2020) **141**(24):e932. doi: 10.1161/CIR.0000000000000876. Erratum in: *Circulation*. (2021) **143**(7):e249. doi: 10.1161/CIR.0000000000000956. 32375490

[97] Joint Commission International. Joint Commission International Accreditation Standards for Hospitals. 8th ed Oak Brook (IL): JCI (2023).

[98] Cohen AT Tapson VF Bergmann JF Goldhaber SZ Kakkar AK Deslandes B Venous thromboembolism risk and prophylaxis in the acute hospital care setting (ENDORSE study): a multinational cross-sectional study. Lancet. (2008) 371(9610):387–94. 10.1016/S0140-6736(08)60202-0 Erratum in: *Lancet*. (2008) **371**(9628):1914. 18242412

[99] Wendelboe AM Langenfeld H Ageno W Castellucci L Cesarman-Maus G Ddungu H Current practices of standardized risk assessment for venous thromboembolism: results from a global survey from the world thrombosis day steering committee. J Thromb Haemost. (2022) 20(2):532–5. 10.1111/jth.15608 34826190

[100] Lam BD Dodge LE Datta S Rosovsky RP Robertson W Lake L Venous thromboembolism prophylaxis for hospitalized adult patients: a survey of US health care providers on attitudes and practices. Res Pract Thromb Haemost. (2023) 7(6):102168. 10.1016/j.rpth.2023.102168 37767063 PMC10520566

[101] Han M Huang J Yang J Chen J Qi H. Barriers and facilitators to the implementation of guidelines for venous thromboembolism prevention and management: a mixed-methods systematic review. Int J Nurs Stud Adv. (2024) 8:100273. 10.1016/j.ijnsa.2024.100273 39717799 PMC11664414

[102] Catterick D Hunt BJ. Impact of the national venous thromboembolism risk assessment tool in secondary care in England: retrospective population-based database study. Blood Coagul Fibrinolysis. (2014) 25(6):571–6. 10.1097/MBC.0000000000000100 24686103 PMC4162339

[103] Bahl V Moote MJ Hu HM Campbell DA Jr. Impact of clinical decision support with mandatory versus voluntary venous thromboembolism risk assessment in hospitalized patients. TH Open. (2024) 8(3):e317–28. 10.1055/s-0044-1790519 39268041 PMC11392591

[104] Cassidy MR Rosenkranz P McAneny D. Reducing postoperative venous thromboembolism complications with a standardized risk-stratified prophylaxis protocol and mobilization program. J Am Coll Surg. (2014) 218(6):1095–104. 10.1016/j.jamcollsurg.2013.12.061 24768293

